# Therapeutic drug monitoring of intravenous amikacin during peritoneal dialysis for *Mycobacterium abscessus* exit-site infection: a case report

**DOI:** 10.1186/s40780-026-00593-z

**Published:** 2026-06-08

**Authors:** Yuki Shimizu, Toshinori Hirai, Ei Kusahana, Kaori Hosonuma, Takeaki Watanabe, Keiko Kadota

**Affiliations:** 1 Department of Pharmacy, Tokyo Dental College Ichikawa General Hospital, 5-11-13 Sugano, Ichikawa, Chiba 272–8513 Japan; 2 Department of Pharmacy, Institute of Science Tokyo Hospital, 1-5-45 Yushima, Bunkyo- ku, Tokyo, 113-8519 Japan; 3 Department of Internal Medicine, Tokyo Dental College Ichikawa General Hospital, 5-11-13 Sugano, Ichikawa, Chiba 272–8513 Japan; 4 Department of Clinical Pharmacy, Tokyo Dental College Ichikawa General Hospital, 5-11-13 Sugano, Ichikawa, Chiba 272–8513 Japan

**Keywords:** Amikacin, Therapeutic drug monitoring, Peritoneal dialysis, Continuous ambulatory peritoneal dialysis

## Abstract

**Background:**

Patients with peritoneal dialysis (PD) often develop exit-site infections (ESI) caused by non*tuberculous mycobacteria*. Antimicrobial therapy, including intravenous amikacin (AMK), is combined with PD catheter removal to control the source of infection. However, the efficiency of AMK removal using PD remains unclear. In this report, we describe AMK pharmacokinetics before and after PD catheter removal in a patient diagnosed with *Mycobacterium abscessus* from ESI.

**Case presentation:**

A 73-year-old female PD patient weighing 42.4 kg who received intravenous AMK + clarithromycin + imipenem/cilastatin for *Mycobacterium abscessus* ESI on day 1 under therapeutic drug monitoring. During hospitalization, the patient underwent a typical PD regimen including nocturnal continuous ambulatory PD with Reguneal HCa 1.5^®^ (1.5% glucose) 1.5 L (2-hour storage) + Reguneal HCa 1.5^®^ 1.5 L or Extraneal^®^ (7.5% icodextrin) 1.5 L (11.5-hour storage). Dosing schedule of AMK was 200 mg every 48 h to maintain an AMK trough concentration around 10 µg/mL. On day 14, the PD catheter was removed and reinserted on the contralateral side for source control of the infection site and PD was discontinued until day 20. The elimination rate constant (Ke) of AMK was 0.027 h^− 1^ obtained from peak concentration (C_peak_) of 16.3 µg/mL on day 1 and trough concentration (C_trough_) of 4.5 µg/mL on day 3. In contrast, Ke was 0.020 h^− 1^ calculated from both C_peak_ of 22.2 µg/mL on day 9 and C_trough_ of 8.4 µg/mL on day 11, as well as C_peak_ of 23.8 µg/mL on day 17 and C_trough_ was 9.3 µg/mL on day 19. Combination antibiotic therapy was completed due to clinical improvement on day 27. Post-treatment, clofazimine and clarithromycin were administered orally, and the patient was discharged on day 32.

**Conclusions:**

The effect of PD on AMK concentration was limited during therapeutic drug monitoring.

## Background

Peritoneal dialysis (PD) is an important renal replacement therapy option. It is preferable in terms of preserving residual renal function, reducing daytime dialysis constraints, and improving quality of life compared with hemodialysis (HD) [1, 2]. However, it poses a risk of infection complications such as exit-site infection (ESI) and peritonitis [3, 4]. These infections are often caused by non*tuberculous mycobacteria* (NTM) and are treated using combination therapies such as amikacin (AMK), a common aminoglycoside antimicrobial agent, clarithromycin (CAM), and imipenem/cilastatin (IPM/CS) [5, 6]. In cases of PD-associated peritonitis, intraperitoneal antibiotic therapy is recommended [4]. On the other hand, intravenous antibiotic therapy is also used for ESI [3, 7].

The efficacy of AMK is associated with the ratio of peak concentration (C_peak_) to the minimum inhibitory concentration (MIC), while side effects such as nephrotoxicity and ototoxicity correlate with its trough concentration (C_trough_) [8–10]. The large variation in AMK clearance (CL) depends on creatinine clearance (CCr) [11]. Therefore, therapeutic drug monitoring (TDM) should be performed in clinical practice [8–10]. Since a relatively high dose is required to attain C_peak_ over the target window, achieving the target peak while avoiding excessive C_trough_ in end-stage renal disease (ESRD) is problematic. Accordingly, aminoglycosides, including AMK, are recommended to be administered as a reduced single dose with a target area under the concentration time curve (AUC)/MIC of 30–50 (non-critically ill immunocompetent patients) and 80–100 (critically ill patients) for gram-negative infections [9, 12].

Treatment of NTM infections, particularly *Mycobacterium abscessus*, is often refractory [13, 14]. Therefore, accurate dosing of antimicrobial agents for each patient is important. However, clinicians have difficulty personalizing AMK dosing for NTM-related ESI infections in PD settings because of the limited information on TDM [15]. In particular, little is known about the AMK removal efficiency in PD. AMK is reported to be approximately 50% removed by HD because its pharmacokinetic (PK) profile includes low protein binding ratio and volume distribution (Vd) (0.25 L/kg) [11, 16]. Regeur et al. [17] clarified that the mean CL increased 2.56-fold from 2.5 to 6.4 mL/min/m^2^, before and after PD. Meanwhile, Madhavan et al. [18] reported that the mean half-life (t_1/2_) in PD was 29 h, which is equivalent to the t_1/2_ in HD patients on non-dialysis days. However, these reports were based on older patients and may differ from the current PD condition.

Here, we report a case of *Mycobacterium abscessus* ESI treated with AMK before and after PD catheter removal.

### Case presentation

A 73-year-old Japanese female patient (body weight, 42.4 kg) had been receiving continuous ambulatory peritoneal dialysis (CAPD) for chronic glomerulonephritis for 2 years. She had multiple pre-existing conditions, including hypertension, hyperuricemia, and ventricular fibrillation secondary to long QT syndrome, requiring an implantable cardioverter-defibrillator. Pus discharge at the exit-site was observed prior to admission (day − 45), and oral amoxicillin/clavulanic acid (500 mg/125 mg once daily) and levofloxacin (250 mg every 48 h) were administered for 5 weeks. Despite treatment, no improvement was observed. Acid-fast bacillus was detected in the pus (day − 3), and polymerase chain reaction testing ruled out *Mycobacterium tuberculosis*, *Mycobacterium avium*, and *Mycobacterium intracellulare* infections. Based on the refractory course, the patient was suspected of having ESI caused by *Mycobacterium abscessus*. Furthermore, the primary physician excluded peritonitis because there was no increase in the PD solution cell count. The patient was hospitalized and received a combination therapy of AMK + CAM + IPM/CS (day 1) [19]. AMK was infused intravenously at an initial dose of 200 mg (4.7 mg/kg) over 30 min, every 48 h, with the first dose given at 2:00 PM. AMK C_peak_ 30 min after administration on day 1 and C_trough_ immediately before administration on day 3 (48 h after the initial dose) were measured. The AMK dosage was adjusted on the basis of TDM results. CAM was administered orally at 200 mg once daily and IPM/CS was administered intravenously at 250 mg twice daily.

The clinical data are shown in Table 1. We calculated the kidney function using estimated glomerular filtration rate (eGFR): eGFR = 194 × serum creatinine^− 1.094^ × age^− 0.287^ × 0.739 (if female) [20] and Cockcroft–Gault (CG) formula: estimated CCr = (140 − age) × bodyweight / (72 × serum creatinine) × 0.85 (if female) [21]. The estimated CG level was less than 10 mL/min, and the urine volume was approximately 1000 mL/day (Table 1). During hospitalization, CAPD was performed for 13.5 h overnight, with PD solutions alternating between continuous Reguneal HCa 1.5^®^ (1.5% glucose) 1.5 L (2 h storage) + Reguneal HCa 1.5^®^ 1.5 L (11.5 h storage) /day and continuous Reguneal HCa 1.5^®^ 1.5 L (2 h storage) + Extraneal^®^ (7.5% icodextrin) 1.5 L (11.5 h storage) /day. The daily fluid removal volume via CAPD was 200–500 mL.

**Table 1:** Clinical laboratory data

	day − 45	day − 31	day − 3	day 6	day 9	day 11	day 15	day 21	day 25	day 29
BUN, mg/dL	73.0	45.7	63.6	78.5	79.8	79.9	−	75.2	71.1	
Serum creatinine, mg/dL	5.16	4.56	4.95	5.54	6.01	6.37	−	6.54	5.46	5.58
eGFR, mL/min/1.73 m^2^	7.0	8.0	7.3	6.4	5.9	5.5	−	5.4	6.5	6.4
eCCr, mL/min	6.5	7.4	6.8	6.1	5.6	5.3	−	5.1	6.1	6.0
WBC, /µL	6100	5300	6500	5200	6000	6000	8000	4900	5500	5600
Neut, %	74.6	69.9	74.6	59.0	64.9	67.4	83.3	55.5	57.4	54.4
CRP, mg/L	0.20	1.17	0.22	0.05	0.03	0.04	1.33	0.49	0.14	0.07
Urine volume, mL/day	−	−	−	1600	500	1000	1350	1500	1300	1200
PD solution cell count, /µL	−	−	3	21	−	−	−	−	−	114

BUN, blood urea nitrogen; eGFR, estimated glomerular filtration rate; eCCr, estimated creatinine clearance; WBC, white blood cell; Neut, neutrophil; CRP, C-reactive protein; PD, peritoneal dialysis

Hospitalization and initiation of antibiotic combination therapy is day 1

Since there was no established target AMK concentration in PD, we alternatively set C_trough_ <10 µg/mL based on the HD setting [8]. Because the patient had ESRD, we assumed that AMK elimination during the 30 min infusion period was negligible. The PK parameters of AMK were calculated using a 1-compartment model as follows:

$$\eqalign{ & Elimination\>rate\>constant\>\left( {Ke} \right)\left( {{h^{ - 1}}} \right) \cr & = {{ln\left( {{C_{peak}}} \right) - ln\left( {{C_{trough}}} \right)} \over {{t_{trough}} - {t_{peak}}}} \cr} $$

$$\:{t}_{\raisebox{1ex}{$1$}\!\left/\:\!\raisebox{-1ex}{$2$}\right.}\:\left(h\right)=\frac{0.693}{Ke}$$

$$\:{C}_{max}\:\left(\mu\:g/mL\right)=\frac{{C}_{peak}}{{e}^{-Ke\times\:{t}_{peak}}}$$

$$\:Vd\:\left(L\right)=\frac{Dose}{{C}_{max}-{C}_{trough}}$$

$$\:CL\:\left(L/h\right)=Vd\times\:Ke$$

where t_trough_ is the time from the end of administration to the measurement of C_trough_, t_peak_ is the time from the end of administration to the measurement of C_peak_, and C_max_ is the predicted AMK concentration immediately after administration. AMK concentrations were then predicted using the following equation.

$$\:{C}_{i}\:\left(\mu\:g/mL\right)=\:{C}_{max}\:\times\:\:{e}^{\left(-Ke\:\times\:\:{t}_{i}\right)}$$

where C_i_ (µg/mL) represents the predicted concentration t_i_ hours after end of infusion. Moreover, we predicted individual C_i_ values every 6 h between 0 and 48 h after AMK administration by substituting C_max_ and Ke as described in detail. Using the obtained C_i_, the AUCs for 0–24 h (AUC_0−24_) and 24–48 h (AUC_24−48_) were estimated using the linear trapezoidal rule.

**Fig. 1:**
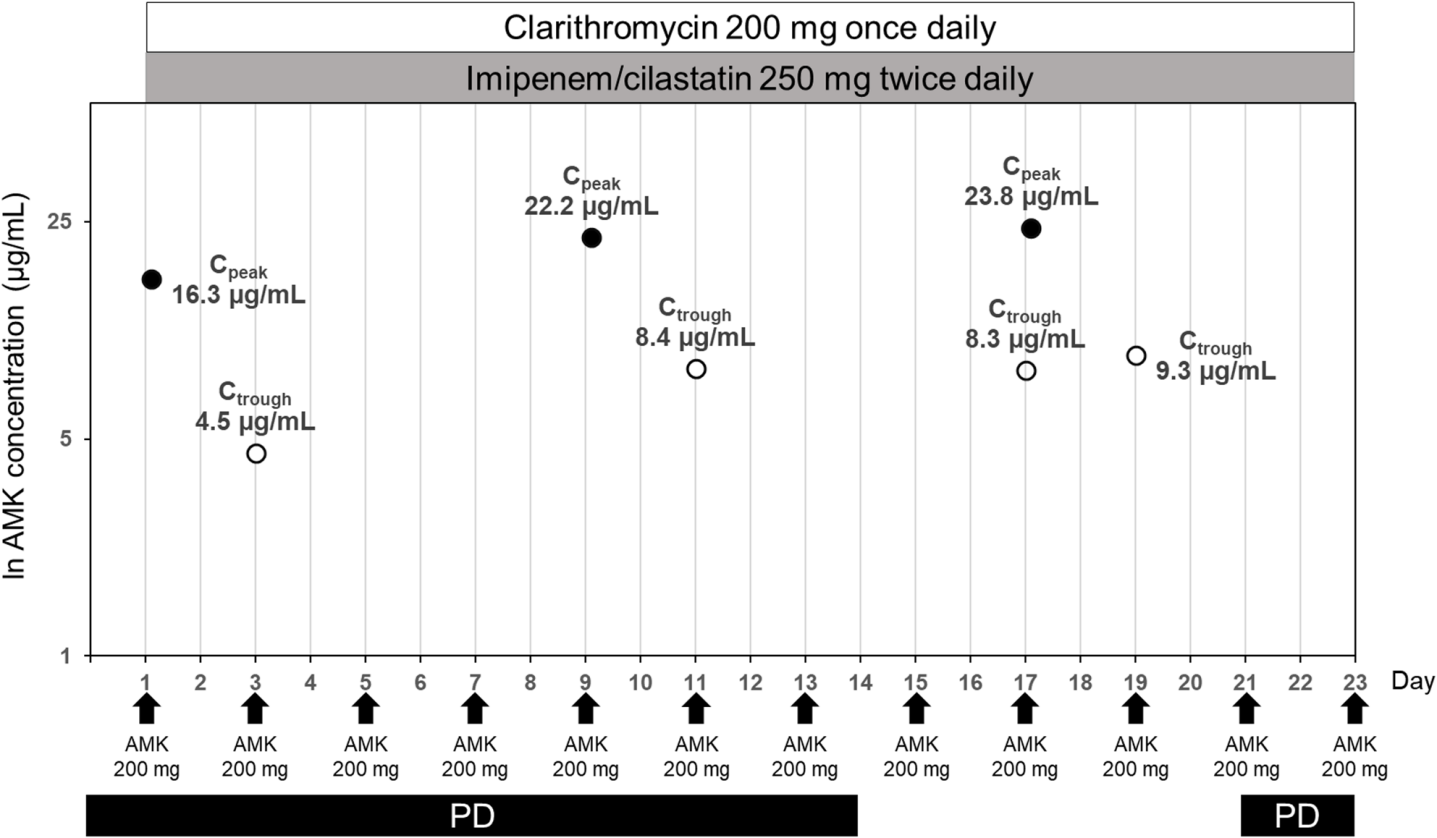
Summary of the patient’s treatment course. X-axis and Y-axis represent days since initiation of amikacin (AMK) administration and AMK concentration in natural logarithm scale. AMK dosage was fixed at 200 mg of 30 min infusion every 48 h. Peritoneal dialysis (PD) was performed from day 1 to 14, and was discontinued from day 15 to 20. Cpeak: black circles, Ctrough: white circles

AMK concentrations were 16.3 µg/mL (C_peak_) on day 1 and 4.5 µg/mL (C_trough_) on day 3 (Fig. 1). PK parameters were as follows: 0.027 h^− 1^ for Ke, 12.1 L for Vd, and 0.33 L/h for CL (Table 2 ). Therefore, we continued administering 200 mg of AMK every 48 h. On day 6, the patient presented with mild dysgeusia (increased bitterness). This event was attributed to a side effect of CAM; therefore, combination antibiotic therapy was continued. Based on the calculations using C_peak_ of 22.2 µg/mL on day 9 and C_trough_ of 8.4 µg/mL on day 11, Ke was 0.020 h^− 1^, t_1/2_ was 34.7 h, AUC_0−24_ was 433.6 mg·h/L, and AUC_24−48_ was 260.6 mg·h/L (Fig. 1; Table 2 ). Thus, AMK reached a steady state thereafter. Although Ke decreased compared with previous measurements, this was considered to be due to decreased renal function, as indicated by the estimated CCr of 5.3 mL/min on day 11 (Table 1).

**Table 2:** Summary of amikacin pharmacokinetic parameters over follow-up period

	day 1-3	day 9-11	day 17-19
Ke, h^−1^	0.027	0.020	0.020
t_1/2_, h	25.7	34.7	34.7
Vd, L	12.1	−	12.7
CL, L/h	0.33	−	0.25
AUC_0−24_, mg·h/L	296.3	433.6	467.9
AUC_24__−48_, mg·h/L	152.5	260.6	286.0

Ke, elimination rate constants; t_1/2_, half-life; Vd, volume of distribution; CL, clearance; AUC_0−24_, area under the concentration time curve from 0 to 24 hours after AMK administration; AUC_24−48_, area under the concentration time curve from 24 to 48 hours after AMK administration

To achieve source control of the infection site, the PD catheter was removed and reinserted on the contralateral side on day 14. The non-PD period ranged from day 14 to day 20. TDM was performed on day 17 (C_trough_ and C_peak_) and day 19 (C_trough_). Ke was 0.020 h^− 1^, when calculated using C_peak_ on day 17 and C_trough_ on day 19; thus, it was equivalent to that in the previous PD period (days 9 − 11) (Fig. 1; Table 2). AUC_0−24_ was 467.9 mg·h/L, and AUC_24−48_ was 286.0 mg·h/L on days 17 − 19 (Table 2).

On day 18, *Mycobacterium abscessus* was identified (MIC, AMK: 8 µg/mL, CAM: 0.12 µg/mL, and IPM/CS: 8 µg/mL) from the sample isolated on day − 3. The patient resumed PD on day 21. Triple antibiotic therapy was completed due to clinical improvement on day 27, and oral clofazimine (100 mg once daily) and CAM (200 mg once daily) were changed thereafter. To calculate the actual CCr, 24-hour urine collection was performed on days 26 − 27 (measured CCr: 5.6 mL/min). On day 32, the patient was discharged on continuous clofazimine and CAM.

## Discussion and conclusions

We describe the pharmacokinetics of AMK with and without PD in a patient undergoing PD for *Mycobacterium abscessus* ESI. The results suggest that the effect of PD on AMK pharmacokinetics was minimal and did not require dose adjustment considering the general PD condition.

Regarding the pharmacokinetics of AMK, the bioavailability of aminoglycoside agents via the intraperitoneal route was reported to be approximately 50% in the non-peritonitis setting and approximately 70% in the peritonitis setting, whereas CL remained largely unchanged [22, 23]. AMK has a low protein-binding fraction and a low Vd, resulting in high permeability through dialysis membranes and a high HD removal rate of approximately 50% [11, 16]. This finding suggests that AMK may be relatively removable via transfer from the blood to the PD solution through the peritoneum.

Interestingly, the present case showed minimal difference in PK parameters between PD statuses. Generally, the maximum PD CL is the volume of PD drainage per unit time. The patient used 3.0 L of PD solution daily, which indicated that the maximum PD CL was 0.125 L/h (= 3.0 L/24 h). This value corresponded to half of the CL in the present patient who did not undergo PD (days 17 − 19) (Table 2). This suggests that PD conditions may contribute to PD CL. Meanwhile, Smeltzer et al. [22] determined the PK parameters in 5 PD patients undergoing AMK using PD solution (2.5% glucose) of 8 L/day, showing that the mean AMK CL was 0.237 L/h. This CL is numerically lower than those in our case presentation with PD period even though the PD volume was higher than our case presentation. This suggests that factors other than PD volume may be influencing CL. Although PD potentially affects AMK concentration, this discrepancy can be explained as follows. The movement of solutes from the blood into the PD solution was delayed as the molecular weight increased. The molecular weight of AMK is 781, which is higher than that of urea (molecular weight: 60) [16]. Urea takes approximately 4–6 h to sufficiently transfer from blood into the PD solution, and AMK is expected to require an even longer time [24]. In a total of 3.0 L of PD solution retained for 13.5 h, 1.5 L was retained for only 2 h whereas the remaining 1.5 L was retained for 11.5 h. Consequently, sufficient transfer of AMK from the blood to the PD solution may not have occurred. Regeur et al. [17] reported that the mean CL increased from 2.5 to 6.4 mL/min/m² before and after PD. However, the PD conditions in this report required a very large amount of dialysate (approximately 60 L every 30 h) [17]. Sakurada et al. [25] reported that the average daily PD volume for Japanese patients was 5.6 L, and approximately 30% patients were ≤ 4.0 L/day. Considering the PD volume used in the patient, the PD volume was within the typical setting. While previous report on the removal of PD in AMK have been documented, condition such as PD volume vary significantly [17]. Our case report demonstrated the clinical course in PD with modern conditions (3.0 L/24 h) and PD washout, and the effect of PD on AMK blood concentrations is limited. Although different PD conditions were not tested, our case provides useful information. Meanwhile, a temporary decrease in urine volume was observed on day 9 during PD, which might decrease AMK elimination (Table 1). However, AMK CL may have remained unchanged because of a remove of AMK during the PD period (day 9–11). On the other hand, a recovered urine volume might counteract a decreased AMK CL due to PD discontinuation (day 17–19). The peritoneal equilibration test helps determine the PD efficiency, which will be useful for designing the dose regimen. Indeed, sufficient fluid was removed; however, we cannot accurately evaluate peritoneal function or the peritoneal permeability of AMK. Since clinical response and target concentrations were obtained without peritoneal equilibration test before infection, peritoneal equilibration test might be a supportive but not essential tool in adjusting dose of AMK in PD patients.

Shulha et al. [5] recommended achieving AMK C_max_ of 35–45 µg/mL for the treatment of NTM. However, this target C_max_ is difficult to achieve in patients with ESRD because AMK cannot be sufficiently eliminated. Meanwhile, Kim et al. [26] reported that in patients with NTM receiving AMK therapy, those with AUC_0−24_ ≥250 mg·h/L had a lower re-admission rate than those with AMK AUC_0−24_ <250 mg·h/L. This target AUC including AUC_0−24_ and AUC_24−48_ ≥250 mg·h/L, can be achieved in patients with ESRD by elevating target C_trough_ less than 10 µg/mL (Table 2). This finding may provide a practical target for clinicians when conducting AMK TDM in PD patients with NTM infection. However, C_trough_ of 10 µg/mL is associated with a risk of further impairing residual renal function in PD patients [8]. In the patient, AMK C_trough_ levels up to 10 µg/mL were allowed to prioritize treatment. In the patient, a temporary reduced urine volume and an increase in serum creatinine were noted after AMK admission (Table 1). Monitoring urine volume and renal function is important for appropriate AMK therapy.

In conclusion, PD status may not affect the pharmacokinetics of AMK under typical PD solution volume conditions. This finding is thought to differ according to detailed conditions such as the amount of PD solution and complications of peritonitis. To manage NTM infections in patients with PD, the AMK dosage should be individualized while allowing a C_trough_ below 10 µg/mL.

## Data Availability

No datasets were generated or analyzed during the current study.

## Abbreviations

PD: Peritoneal dialysis
HD: Hemodialysis
ESI: Exit-site infection
NTM: Nontuberculous mycobacteria
AMK: Amikacin
CAM: Clarithromycin
IPM/CS: Imipenem/cilastatin
C_peak_: Peak concentration
MIC: Minimum inhibitory concentration
C_trough_: Trough concentration
CL: Clearance
CCr: Creatinine clearance
TDM: Therapeutic drug monitoring
ESRD: End-stage renal disease
AUC: Area under the concentration time curve
PK: Pharmacokinetic
Vd: Volume of distribution
t_1/2_: Half-life
CAPD: Continuous ambulatory peritoneal dialysis
eGFR: Estimated glomerular filtration rate
CG: Cockcroft–Gault
